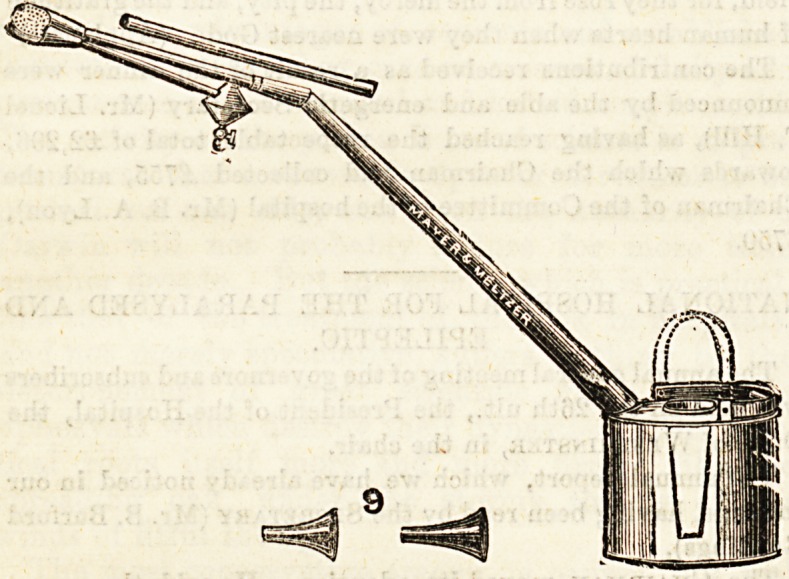# Bronchitis or Tracheotomy Kettle

**Published:** 1892-06-04

**Authors:** 


### FURNITURE AND FITTINGS.

### BRONCHITIS OR TRACHEOTOMY KETTLE.

Our illustration shows an excellent kettle supplied by Messrs. Mayer and Meltzer, and is the invention of Mr. Newton H. Nixon, Secretary of University College Hospital. This kettle does away with the necessity for waterproof sheeting over the bed, as it is designed to carry off the condensed steam.

No lid is required. The kettle is heated over a gas-ring, and not on the fire, although it can be used in either way. It can be placed in any part of a ward or room by regulating the length of india-rubber tubing. The kettle can be adapted for use by either adults or children as the spout is made in sections.

## Figures and Tables

**Figure f1:**